# “Wrinkles will only go where the smiles have been”: a path analysis of the associations between happiness and health, sleep, physical activities for older people in Abu Dhabi

**DOI:** 10.1186/s12877-023-04244-y

**Published:** 2023-09-12

**Authors:** Masood Badri, Mugheer Alkhaili, Hamad Aldhaheri, Guang Yang, Saad Yaaqeib, Muna Albahar, Asma Alrashdi

**Affiliations:** 1 United Arab Emirates University, Al Ain, United Arab Emirates; 2 Department of Community Development, United Arab Emirates University, Al Ain, United Arab Emirates

**Keywords:** Happiness, Sleep, Sport, Health, Older adults, Path analysis, Abu Dhabi

## Abstract

**Background:**

The study aimed to identify the associations of happiness and factors related to physical and mental health, leisure, and sports activities amongst older adults in Abu Dhabi. The sample comprised 1,004 participants in the third Abu Dhabi Quality of Life survey administered in 2019–2020.

**Methods:**

The analysis used path analysis to develop a model incorporating the specified variables. The path model highlighted all direct and indirect associations between the variables. We also used variance analysis to test the differences in gender, marital status, and education attainment with happiness.

**Results:**

Results show that sleep quality is most associated with happiness and subjective health. In addition, sleeping hours did not show any association with subjective health; but were associated with happiness. The result also confirms that mental health is negatively associated with happiness and subjective health. How often an elderly gets involved in sport and activities for at least 30 min significantly affects subjective health and happiness.

**Conclusions:**

Happiness of older adults is best understood when we look at both direct and indirect effects using a path model. Their happiness is significantly associated with their subjective health, mental health, participation in sport and activities and sleep quality, Implications of the study were highlighted, along with future research directions.

## Introduction

The happiness of older adults has taken center stage in many countries as they tend to show more vulnerability considering the critical quality-of-life determinants. Happiness, as a personal state of feelings of enjoyment and satisfaction, reflects the overall subjective well-being of an individual [1, 2]. Over the years, many theorists have studied and described concepts of happiness and subjective well-being [1, 3, 4]. Many scholars argue that people perceive happiness differently and people may be unhappy in certain settings [5–7].

Like others, older adults’ happiness is influenced by various factors, ranging from individual characteristics, health status, mental health, social connection, to day-to-day activities [8–10]. Among these, health conditions, physical activities, and sleep appear to be the most common and essential factors for older people.

The interconnection of health and well-being in general and happiness in particular is stressed by various studies [11–14]. Both subjective physical health and mental health have received much attention as significant determinants of older people’s happiness [15, 16]. A number of international empirical studies have concentrated on subjective indexes such as happiness, self-reported health status, and health conditions and most have reported significantly high correlations between them [17–19].

Sports activities play a significant role in the health of the elderly. Research evidence suggests that participation in physical activity improves older adults’ physical and mental health [20–23]. Research also reports the association of sport and physical activity with increases in subjective well-being, such as happiness and life satisfaction [24–26].

The effect of sleeping hours or sleep duration on happiness has been another focus in the literature [27–29]. Some studies found that short sleep duration is associated with lower happiness in healthy adults [30], while some longitudinal studies have revealed that happiness negatively correlates with sleep duration [31]. Therefore, some researchers argue that more research is needed to explore the associations between sleep duration and happiness, considering other factors such as age [27]. Sleep quality among older people is essential, as the National Institutes of Health [32] reported that as older adults age, the “depth and length of sleep” decreases. Studies hint that older adults become more susceptible to sleep disorders, which is independently and strongly associated with the deterioration of subjective wellbeing [33]. In general, various studies concerning the relationship between sleep quality and wellbeing/happiness have shown that low sleep quality is associated with worse health and wellbeing [30] and the same association is found among the older adults population [10, 34].

Several demographic variables are found to have confound with the relationships between subjective health, mental feelings, sleep duration and quality, and happiness. These factors include age [33], gender [10, 35, 36], marital status [37, 38], and education [34]. In general, most of the applied research yielded similar results on the influence of those bio-variables on older adults.

In the context of Abu Dhabi, [39] found that the happiness of older adults was associated with many variables in the psycho-socio system, including subjective health and mental health. A study utilized hierarchical regression of wellbeing and self-rated health among older adults in Abu Dhabi [40]. Amongst many other variables, subjective health and psychological feelings proved highly significant. Another study [41] focused on retirees’ wellbeing and the results pointed to subjective health as a major significant factor. Considering the findings in the international literature, older adults may be inclined to participate in regular sports and physical activities. In Abu Dhabi, many public entities have designed programs encouraging them to become more active [42]. Nevertheless, the Third cycle of Abu Dhabi Quality of Life survey reported that while 13.2% of adults older than 60 participated in physical activity daily and 25.5% on most days of the week, 22.6% reported rarely and 12.1% never participated in sports activities.

The current study aims to fill the gap in the Abu Dhabi-specific research literature by examining the association between happiness, subjective health status and other well-being determinants in the life of older people aged 60 or above. Specifically, this study investigates how the frequency of physical activity, sleeping hours, quality of sleep, and mental health are associated with older adults’ self-rated physical health and happiness. The results could provide additional insights for policymakers in Abu Dhabi to examine the synergistic combinations of factors associated with older adults’ happiness and health.

## Materials and methods

### Study sample and data collection procedures

The data for this study were obtained from the Third cycle of Abu Dhabi Quality of Life survey (QoL-3). In cooperation with the Abu Dhabi Statistics Center (SCAD), a representative survey of Abu Dhabi community members in all three regions (Abu Dhabi, Al Ain, and Al Dhafra) was conducted by the Department of Community Development (DCD), with assistance of other public and private entities in Abu Dhabi. More specifically, the sample population consisted of both Emiratis and non-Emirati residents aged over 15 years and older. The initial sample size was 82,000, with a sampling error range of 4–5% and a confidence level of 95%. With such criteria, sufficient sample size and statistical power were achieved to reduce potential sampling bias. A total of 6,879 older adults (age above 60) participated in the survey. The survey was reviewed and approved by SCAD and the DCD. The participants were fully informed about the contents of the present study, its objective, and its procedures. In the development and administration of the survey, there were a number of procedures undertaken to reduce potential method bias as recommended by Podsakoff et al. [43]. First, the survey questions adopted varied response formats (e.g. Likert scales, 0–10 point scales) to reduce common scale properties; it was administered through multiple media channels like SMS messages, organizational Emails, and face-to-face interviews with the assistance of enumerators from SCAD; and it was collected from all districts within the Emirate of Abu Dhabi. Second, the survey underwent several official revisions and iterations between SCAD and DCD to ensure item clarity and prevent ambiguity. Third, the survey adopted a modular presentation format where each topic was allocated within an independent ‘block’, which contributes to creating a “proximal or psychological separation” that serves to reduce bias in reporting, as previous responses are less available to the participant which reduces the likelihood of using prior responses to answer subsequent questions, as suggested by Podsakoff et al. [43]. Furthermore, the survey did not require any identifying information, and preserved participants’ anonymity.

### The variables

International studies used diverse concepts and measurements of happiness. Some, such as the Oxford Happiness Inventory, used multiple variables [44] or specifically designed life satisfaction scales [45]. However, most studies adopted a single-item question to measure happiness [24, 45–47]. Many studies have reported this single scale to possess high validity and reliability [14, 48]. In QoL-3, happiness was measured using a single-item question and scored on a 0–10-point scale. The same scale was also used in many international surveys related to quality of life.

Through an open question, the participants were asked to state, “How many hours do you sleep every day?”. They were also asked to describe the quality of their sleep: “How do you rate the quality of your sleep at night?” and five choices were provided (very bad, bad, neither well nor bad, well, and very well). Another question asked the participants to rate their health status: “In general, how do you personally assess your current health?” and the responses were recorded on a five point scale (poor, fair, good, very good, and excellent). The survey measured the mental health of participants through self-assessed eight mental problems or symptoms (sadness, anxiety, concentration, sleeping, stress, fear, loneliness, and boredom) measured on a 5-point frequency scale ranging from “not at all” to “all the time”. These items were composed of a single composite variable – subjective mental health, where a higher value reflected a more negative outcome. This composite variable had a Cronbach Alpha reliability coefficient of 0.928. Other demographic questions included gender, educational attainment, marital status, and region. We normalized the data for the final path analysis.

### Methods of analysis

The current research used path analysis as the most appropriate analytical framework. Theoretically, path analysis envisions specific relationships among all the independent variables. Path analysis framework clarifies how moderator variables influence the paths that constitute the direct, indirect, and total effects of mediated models, which indicate the hypothesized relationships between the variables. In particular, the literature indicates that relationships between psychological (e.g. mental health) and behavioral (e.g. exercise or sleep) are scarcely bivariate, and requires a multivariate approach to capture the accurately capture the associations [49]. Thus, the choice of a path analysis seemed justified. In this study we present an analytical framework for such moderations and mediations for the dependent variable - happiness of older adults. A final path model could also determine the path coefficients’ statistical significance.

A step-by-step path analysis was followed, which led us to develop the optimal path model for the study. We used many fit statistics offered by LISREL. The fit statistics used included the Degrees of Freedom, the Maximum Likelihood Ratio Chi-Square, the P-Value for the Test of Close Fit, the Root Mean Square Error of Approximation (RMSEA), the Comparative Fit Index (CFI), the Normed Fit Index (NFI), the Non-Normed Fit Index (NNFI), the Goodness of Fit Index (GFI), the Adjusted Goodness of Fit Index (AGFI), and the Root Mean Square Residual (RMR).

## Results

### Descriptive statistics

Table 1 shows the profile of respondents in the survey. The majority of the older adults in the survey were males (83.1%) and married (88.2%). About one third (30.7%) of them held a bachelor’s degree, with 15.3% holding only a secondary school certificate. Regarding age distribution, the category of 60–65 represented the largest (55.4%) age group, followed by the 66–70 age group (22.2%). Emiratis made up the majority (74.9%). Two thirds of the respondents (66.6%) lived in the Abu Dhabi region.

**Table 1:** Profile of respondents

	Percentage
**Gender**	
Male	83.1%
Female	16.9%
**Marital status**	
Married	88.2%
Single	2.4%
Divorced	4.1%
Separated	0.9%
Widowed	4.4%
**Education level**	
Illiterate	6.3%
Below secondary school	9.3%
Secondary school	15.3%
Post high school training certificate	5.8%
College diploma	9.2%
Bachelor’s degree	30.7%
Master’s degree	12.5%
Doctorate degree	10.9%
**Age**	
60–65	55.4%
66–70	22.2%
71–75	10.2%
76–80	6.3%
81–85	3.1%
86–90	1.8%
91+	1.0%
**Nationality**	
Emirati	74.9%
Non-Emirati	25.1%
**Region of residence**	
Abu Dhabi	66.6%
Al Ain	22.9%
Al Dhafra	10.5%

Table 2 provides the mean and standard deviations of the six variables used in the model. Happiness scored a mean of 8.347 with a standard deviation of 1.869. For subjective physical health, the mean was 3.381 and the standard deviation was 0.878. Sleeping hours had a mean of 7.216 with a standard deviation of 1.375. Sleeping quality resulted in a mean of 3.865 with a standard deviation of 0.765. The composite variable of subjective mental health produced a mean of 1.650 with a standard deviation of 0.814.

**Table 2:** Descriptive results of variables in the model

Variables	Means	Standard deviations
Happiness	8.347^a^	1.869
Subjective physical health	3.381^a^	0.878
Subjective mental health	1.650 ^a^	0.814
Sleeping hours	7.216 ^a^	1.375
Sleeping quality	3.865 ^a^	0.765
Exercising frequency	3.642 ^a^	1.647

^a^ Higher numbers indicate higher values on the construct.

### Correlations and covariances

Table 3 shows the correlation matrix of all variables considered for the path model. Almost all coefficients are significant at the 0.01 level. Focusing on both happiness and subjective health, the most significant coefficients were sleeping quality and mental health. A high correlation between happiness and subjective health was also revealed. On the other hand, mental health provided negative correlations with all other variables in the model. We should note that exercising was negatively correlated with sleeping hours.

**Table 3:** The correlation matrix

	SH	HP	SH	SQ	MH	OE
Subjective Health (SH)	1	0.262*	0.029	0.353*	-0.233*	0.191*
Happiness (HP)		1	0.181*	0.387*	-0.310*	0.123*
Sleeping Hours (SH)			1	0.281*	-0.140*	-0.085*
Sleeping Quality (SQ)				1	-0.385*	0.086*
Mental Health (MH)					1	0.090*
Often Exercise (OE)						1

* significant at p ≤ 0.01

The analysis used covariances in designing feasible path analyses that reflect how two random variables in a data set would change together (Lu et al., 2019). In addition, path analysis utilizes the correlation (covariance) between two variables to express the sum of the compound paths connecting them (Garre-Olmo et al., 2017). Table 4 shows the covariance matrix, where the covariance scores indicate the relationship of two variables when one variable changes.

**Table 4:** The covariance matrix

	SH	HP	SH	SQ	MH	OE
Subjective Health (SH)	0.524					
Happiness (HP)	0.632	8.543				
Sleeping Hours (SH)	0.106	1.070	3.013			
Sleeping Quality (SQ)	0.150	0.768	0.308	0.360		
Mental Health (MH)	-0.088	-0.643	-0.136	-0.127	0.338	
Often Exercise (OE)	0.043	0.336	0.026	0.019	-0.020	-0.020

### Path analysis results

**Fig. 1:**
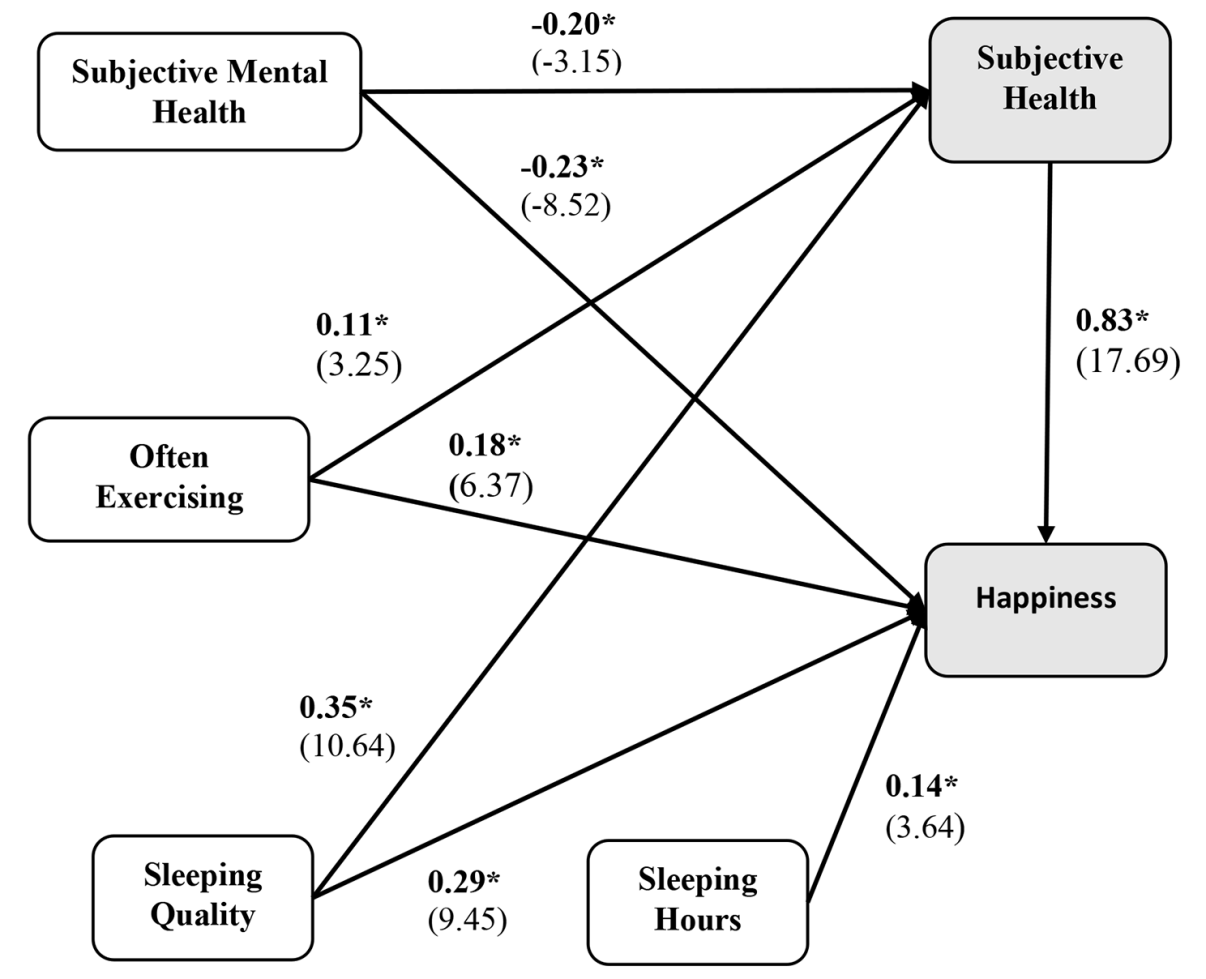
Path model of sleep quality and well-being factors for working adults. A graphical representation of the specified model of sleep quality and well-being factors for working adults. Each path is labeled with the estimate (in bold), with the t-values indicated between brackets. *p ≤ 0.05

As shown in Table 5, the final path model produced an RMSEA of 0.0420 with all fit indexes being above the thresholds recommended by experts (the minimum being 0.996 for the AGFI). Therefore, a model that incorporates a total of six factors was produced (Fig. 1). Table 6 summarizes the path coefficients and their associated significance values. The highest significant estimate is associated with subjective health and happiness (0.83 with t = 17.96). Other higher estimates are shown between sleeping quality and subjective health (t = 10.64). Only mental health shows a negative association with subjective health and happiness.

**Table 5:** Goodness-of-fit statistics

Degrees of Freedom	1
Maximum Likelihood Ratio Chi-Square	0.644 (P = 0.4223)
Root Mean Square Error of Approximation (RMSEA)	0.0420
Normed Fit Index (NFI)	0.999
Non-Normed Fit Index (NNFI)	0.999
Comparative Fit Index (CFI)	0.999
Incremental Fit Index (IFI)	0.999
Root Mean Square Residual (RMR)	0.00671
Goodness of Fit Index (GFI)	0.999
Adjusted Goodness of Fit Index (AGFI)	0.996

**Table 6:** Final model path estimates and t-values

From	To	Estimate	t-value	Significance
Subjective mental health	Subjective health	-0.20	-3.15	0.001
Often exercising	Subjective health	0.11	3.25	0.001
Sleeping quality	Subjective health	0.35	10.64	0.001
Subjective health	Happiness	0.83	17.69	0.001
Often exercising	Happiness	0.18	6.37	0.001
Sleeping quality	Happiness	0.29	9.45	0.001
Sleeping hours	Happiness	0.14	3.64	0.001
Subjective mental health	Happiness	-0.23	-8.52	0.001

Table 7 shows the direct, indirect, and total associations between all variables and happiness and subjective health. The results show that three variables - subjective mental health, often exercising, and sleeping quality had direct and indirect effects on happiness. In addition, the highest total effect is between subjective health and happiness (0.83) and between sleep quality and happiness (0.58). Subjective mental health also has a significantly high total effect on happiness (0.40), highlighting that the subjective mental health of older people should receive close attention.

**Table 7:** Final model path direct and indirect effects

From	To	Direct effect	Indirect effect	Total effect
Subjective mental health	Subjective health	-0.20	----	-0.20
Often exercising	Subjective health	0.11	----	0.11
Sleeping quality	Subjective health	0.35	----	0.35
Subjective health	Happiness	0.83	----	0.83
Often exercising	Happiness	0.18	0.09	0.27
Sleeping quality	Happiness	0.29	0.29	0.58
Sleeping hours	Happiness	0.14	----	0.14
Subjective mental health	Happiness	-0.23	0.17	0.40

It is essential to shed light on the differences in the presented results according to specific categories of respondents (i.e., gender, age, marital status, education level, region). However, we need to be cautious that there are noticeable differences in the number of respondents in each different segmentation. The results of further analysis of variance (ANOVA) show some significant differences among different categories of respondents as follows.

Emiratis reported a much higher score for happiness than non-Emiratis (8.68 relative to 8.22). Male older adults reported a significantly higher mean than females (8.39 relative to 8.07). We did not observe significant differences among older adults of different marital status.

For subjective health, there are significant differences across marital status and nationality categories. The divorced reported the highest subjective health score (3.73), while the single recorded the lowest mean of 3.30. Meanwhile, Emiratis reported a mean of 3.56, compared to 3.32 for non-Emiratis.

For subjective mental health, significant differences were noted among older adults of different marital status, gender, educational attainment, and nationality. The married reported the best outcome (1.96), while the separated reported the worst (2.70). Non-Emiratis reported a much higher mean concern than Emiratis (2.12 vs. 1.82). Females reported more negative mental health than males (2.28 relative to 1.97). Those with doctorate degrees reported the highest mean concern, while those with secondary education reported the most positive mean (2.29 relative to 1.72).

For sleep quality, the married enjoyed the best sleep quality, while the single reported the worst (3.90 relative to 3.35). Emiratis reported the highest mean compared to non-Emiratis (4.05 relative to 3.79). Male respondents reported a significantly higher mean than females (3.88 relative to 3.74). Those with primary school education reported a higher mean (4.14) than that of holders of post high school education attainment (3.80).

For sleeping hours, we observe no differences between Emiratis and non-Emiratis, between region of residence, and between marital status. However, there was a significant difference between males (3.69) and females (3.38). Significant differences were also recorded among older adults of different education attainment, as those with a master’s degree or PhD scored the highest (4.13), while those with only read and write (2.53) and primary school education (3.20) scored the lowest.

For frequency of doing sports, there was no differences between Emiratis and non-Emiratis, between marital status and region of living in Abu Dhabi. However, older male people scored significantly higher (3.70) than females (3.36). Meanwhile, those with a PhD (4.25) or a master’s degree (4.16) scored significantly higher than those with only read and write (2.51) and those with primary school education (3.22).

## Discussions

The study produced a path model with significant fit statistics, confirming the importance of several determinants of happiness for old people in Abu Dhabi. These happiness determinants were physical health, mental health, sleep quality and duration, and the frequency of participating in sports activities.

The association of subjective physical health with happiness is significant in the Abu Dhabi study. For Abu Dhabi, older adults’ highest significant path estimate is observed between subjective health and happiness. Consistently, in many studies of different cultures as well, happiness is reflected in terms of subjective health [5, 11, 50]. Moreover, this study confirms the strong negative association between mental health and happiness, supporting various studies that indicate the vital role of mental or psychological feelings in the life of elderly [12, 13, 51].

The path model results in a highly optimistic estimate on the effects of sleep quality and duration on older adults and their happiness. The results are consistent with other international research focusing on the associations between sleeping hours or sleep duration and happiness [27, 29, 30]. Apart from a direct association between sleeping quality and happiness, the path model also reveals a strong indirect association between sleeping quality and happiness through subjective health. These estimates are both theoretically and empirically sound, showing that good sleep quality is significantly associated with older adults’ health and overall wellbeing [52, 53].

A direct effect of the frequency of participating in sports activities on happiness and subjective health, as well as an indirect effect on happiness, proves again the positive relationships between practicing sports, health, and happiness [9, 10, 52]. As people age, health has become the most significant factor affecting the life of older adults and their life satisfaction and happiness and participation in sports and physical exercises has various significant mental and physical health benefits [20, 23, 54] Growing and consistent evidence on the positive impact of sports and activities on the health and well-being of older adults has led to the design and implementation of sports-focused policies to enhance the quality of life of older people worldwide [22].

Having confirmed the importance of physical and mental health, sleep quality and duration, and participation in sports activities for older adults, we should note that significant differences exist across different segments of the older adult population, as revealed by this Abu Dhabi study. For example, male older adults in Abu Dhabi tended to report higher happiness scores, enjoyed longer sleeping hours, better sleep quality, and more positive subjective health. While this is consistent with the results of other studies [27, 55], there appears to be gendered health and well-being outcomes resulted from socio-economic and cultural structures [56]. Education attainment also played a role in sleep duration and the frequency of taking sports activities, as those with lower education recorded the lowest subjective mental feelings. Results are consistent with other studies that note that higher education levels are significantly associated with better mental health [37]. These health and well-being differences among different groups of older adults should be fully noted and could help policymaker better design policies aimed at enhancing older adults’ health status and well-being [17].

### Theoretical and practical implications

The study highlights the significant interconnections between happiness and various factors related to physical and mental health, leisure, and sports activities among older adults in Abu Dhabi. This emphasizes the need to consider multiple determinants of well-being when studying happiness in older populations. In particular, the study’s findings provides support for integrated and wholistic theories of wellbeing, such as Wilson & Cleary’s Quality of Life model [57] which proposes a multi-layered approach to wellbeing involving psychological, social, economic, biological, and environmental factors. This approach is still gaining momentum with revised models [58], and the current study further supports its theoretical foundations between health elements and overall quality of life among older adults. Furthermore, the study findings lends support to models associating sleep quality with happiness and better overall quality of life [59], as well as models of happiness based on physical exercise factors [60], such as the RICH theory of Happiness [61] that focuses on physical health as a precursor to achieving happiness. The findings also contribute towards validating behavioral change models such as the Healthy Ageing Model [62] that involves health coaching and counseling to increase healthy habits such as sufficient sleep hours and physical activities.

There are several practical implications for consideration. For example, health practitioners should recognize the importance of analyzing the results according to specific categories of respondents, such as gender, age, marital status, education level, and region. This can provide valuable insights into the differences and specific needs of different groups of older adults. Additionally, policymakers should be aware of the significant differences in health and well-being outcomes among different groups of older adults. For example, as mentioned earlier male older adults in Abu Dhabi tend to report higher happiness scores, better sleep quality, and more positive subjective health. These differences should be taken into account when designing policies and interventions to enhance older adults’ health and well-being, and female older adults may be eligible as a priority beneficiary of such interventions.

### Limitations

In this study, there are a number of limitations that should be taken into consideration before interpreting the results presented. First, the study design is cross-sectional, and therefore it is not possible to determine causal relationships between the variables in the model. Second, the measurement of the variables depended on a self-reported survey, where individuals may over-report or under-report certain variables due to social desirability bias [63]. Third, the ethnic composition of the sample (75% Nationals vs. 25% Non-Nationals) may call for a separate analysis for each group, as wellbeing models were shown to differ across cultures [64–66]. Therefore, the model fit may vary across cultural groups leading to alternate practical implications. Finally, while the variables in this study were mainly operationalized using single measures as justified in the methodology due to evidence of adequate reliability and validity [14, 48], the use of established psychosocial scales to operationalize each variable may offer a more robust approach to investigate psychological factors associated with wellbeing.

## Conclusions

This study has interrogated the perceptions of happiness, subjective health, mental health, sleeping quality and duration, and physical exercises among older adults in Abu Dhabi. The interconnections of these determinants are found to be significant. The results show that the perception of a happy and fulfilling old age in Abu Dhabi seems to reflect through such interrelated wellbeing determinants.

The path model results provide more inclusive definitions and associates of happier aging that could benefit social policymakers, healthcare professionals, and researchers by paying close attention to the aging individuals’ demands and expectations for happy aging. The vital efforts to design appropriate strategies for maintaining and boosting older people’s physical and mental health should be recognized. Specifically, sport-based social capital intervention could be prioritized to increase older adults’ happiness for successful aging. Concerned authorities, health practitioners, and activity professionals and therapists in touch with older adults should actively encourage them to participate in regular physical activities.

It is worth emphasizing that Abu Dhabi has created the Well-being Committee that reports directly to the Executive Council. The committee shall supervise and follow up on the indicators of quality of life within the Emirate of Abu Dhabi. Such a high-level initiative sends a signal to all Abu Dhabi communities that all needed plans and policies to improve the quality of life will be adopted and implemented, which this study could contribute to.

## Data Availability

The datasets used and analyzed during the current study are available from the corresponding author on reasonable request.
